# The small molecule ZY-214-4 may reduce the virulence of *Staphylococcus aureus* by inhibiting pigment production

**DOI:** 10.1186/s12866-021-02113-5

**Published:** 2021-02-27

**Authors:** Jingyi Yu, Lulin Rao, Lingling Zhan, Bingjie Wang, Qing Zhan, Yanlei Xu, Huilin Zhao, Xinyi Wang, Yan Zhou, Yinjuan Guo, Xiaocui Wu, Zengqiang Song, Fangyou Yu

**Affiliations:** 1 Department of Laboratory Medicine, The First Affiliated Hospital of Wenzhou Medical University, Wenzhou, 325000 China; 2 Department of Clinical Laboratory, Shanghai Pulmonary Hospital, Tongji University School of Medicine, Shanghai, 200082 China; 3 Nanchang University, Nanchang, 330027 China; 4 School of Pharmaceutical Sciences, Wenzhou Medical University, Wenzhou, 325000 China; 5 Shanghai Key Laboratory of Tuberculosis, Shanghai Pulmonary Hospital, Tongji University School of Medicine, Shanghai, 200082 China

**Keywords:** *Staphylococcus aureus*, Pigment, *crtM*, *Sod*, Oxidation

## Abstract

**Background:**

In recent years, clinical *Staphylococcus aureus* isolates have become highly resistant to antibiotics, which has raised concerns about the ability to control infections by these organisms. The aim of this study was to clarify the effect of a new small molecule, ZY-214-4 (C_19_H_11_BrNO_4_), on *S. aureus* pigment production.

**Results:**

At the concentration of 4 μg/mL, ZY-214-4 exerted a significant inhibitory effect on *S. aureus* pigment synthesis, without affecting its growth or inducing a toxic effect on the silkworm. An oxidant sensitivity test and a whole-blood killing test indicated that the *S. aureus* survival rate decreased significantly with ZY-214-4 treatment. Additionally, ZY-214-4 administration significantly reduced the expression of a pigment synthesis-related gene (*crtM*) and the superoxide dismutase genes (*sodA*) as determined by real-time quantitative polymerase chain reaction (RT-qPCR) analysis. ZY-214-4 treatment also improved the survival rate of *S. aureus*-infected silkworm larvae.

**Conclusions:**

The small molecule ZY-214-4 has potential for the prevention of *S. aureus* infections by reducing the virulence associated with this bacterium.

**Supplementary Information:**

The online version contains supplementary material available at 10.1186/s12866-021-02113-5.

## Background

The skin and nasopharynx of approximately 20 to 30% of the world’s population [1, 2] are continuously colonized by the *Staphylococcus aureus*. This bacterium is an opportunistic pathogen that can cause superficial skin diseases and numerous fatal diseases such as bacteremia and infective endocarditis, and also causing osteoarticular, pleuropulmonary, and device-related infections [3–6]. Vancomycin, a glycopeptide antibiotic that can inhibit cell wall biosynthesis, is the first-choice treatment for methicillin-resistant *S. aureus* (MRSA) infections [7, 8]; however, moderate or complete resistance to this antibiotic has become widespread among *S. aureus* strains [8, 9]. Importantly, although significantly fewer antibiotics have been identified or synthesized this century compared with the last century [10], the prescription of antibiotics for the treatment of infections over the years has led to the emergence of drug-resistant *S. aureus* strains [11]. Eliminating bacterial virulence factors is increasingly used as a means of combating antibiotic resistance [12], and represents a strategy that avoids the emergence of drug resistance induced by bacterial stress [12, 13].

Notably, the success of *S. aureus* as a pathogen also lies in its ability to reduce oxidative stress [14]. Superoxide dismutase (SOD) is a key detoxifying enzyme [14–16] that converts reactive oxygen species (ROS) into less harmful products, thereby allowing bacteria that infect the body to escape the body’s immune system and survive [12, 14]. Pigments produced by pathogenic microbes are known to be important virulence factors [17]. *S. aureus* defective in pigment production exhibit reduced infectivity and increased vulnerability to neutrophils [18], and cannot infect the mice in the mouse model [3, 19]. For example, *S. aureus* mutants with defective carotenoid biosynthesis are more likely to be killed by oxidants, show impaired neutrophil survival and lower pathogenicity [20]. Pigment biosynthesis is mediated by proteins encoded by a five-gene cluster (*crtM*, *crtN*, *crtP*, *crtQ*, and *crtO*) [21], which represents a potential new target for antibacterial therapy.

ZY-214-4, molecular formula C_19_H_10_BrNO_4_, contains a chromone ring and an *N*-phenyl-substituted maleimide. Chromone and its derivatives are widely distributed in naturally occurring products and pharmaceuticals as key scaffolds, and chromone derivatives have been shown to exert antimicrobial activities against *Penicillium* spp., *Escherichia coli*, and *Shigella flexneri* [22–24]. Maleimide motifs are prevalent in many natural products and drug candidates, and possess a broad spectrum of biological properties, including antitumor and antibacterial activities [25–27]. However, no studies have reported on the antibacterial activity of chromone–maleimide hybrids in inhibiting golden pigment production in *S. aureus*. In this study, we sought to clarify whether subinhibitory concentrations of ZY-214-4 can inhibit pigment production in clinical *S. aureus* strains.

## Results

### The effect of subinhibitory concentrations of ZY-214-4 on the growth of *S. aureus* strains

**Fig. 1:**
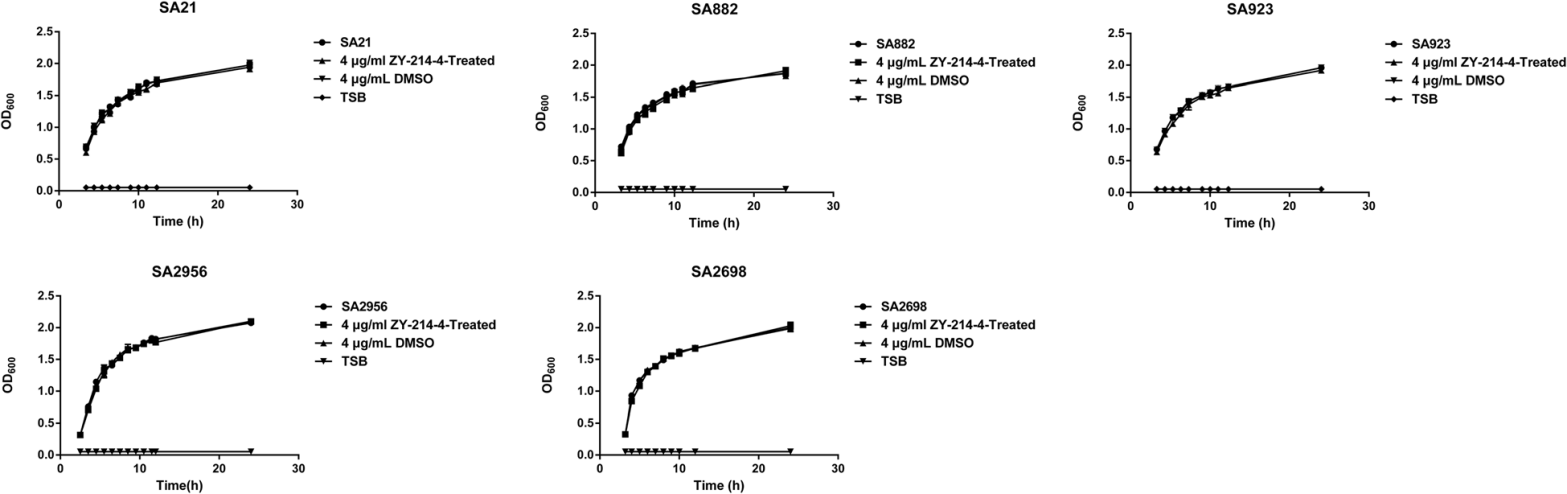
Growth curves for *Staphylococcus aureus* strains cultured with ZY-214-4. TSB was used as a blank control. Images made by GraphPad Prism 6 (GraphPad Software, version 6.00, https://www.graphpad.com/)

The minimum inhibitory concentration (MIC) of ZY-214-4 was 64 μg/mL against *S. aureus* strains SA21, SA882, and SA923, and 256 μg/mL against strains SA2698 and SA2956. To verify whether ZY-214-4 reduced the virulence of *S. aureus* by reducing the expression of virulence genes rather than the number of *S. aureus* cells, we generated a growth curve for these clinical isolates of *S. aureus* at a series of subinhibitory concentration **(Additional** Figure 1**)**. We found that the number of bacteria in the late logarithmic growth period remained constant at the subinhibitory concentration of 4 μg/mL of ZY-214-4 **(**Fig. 1**)**. Therefore, this concentration was used for subsequent experiments.

### ZY-214-4 inhibited pigment production

**Fig. 2:**
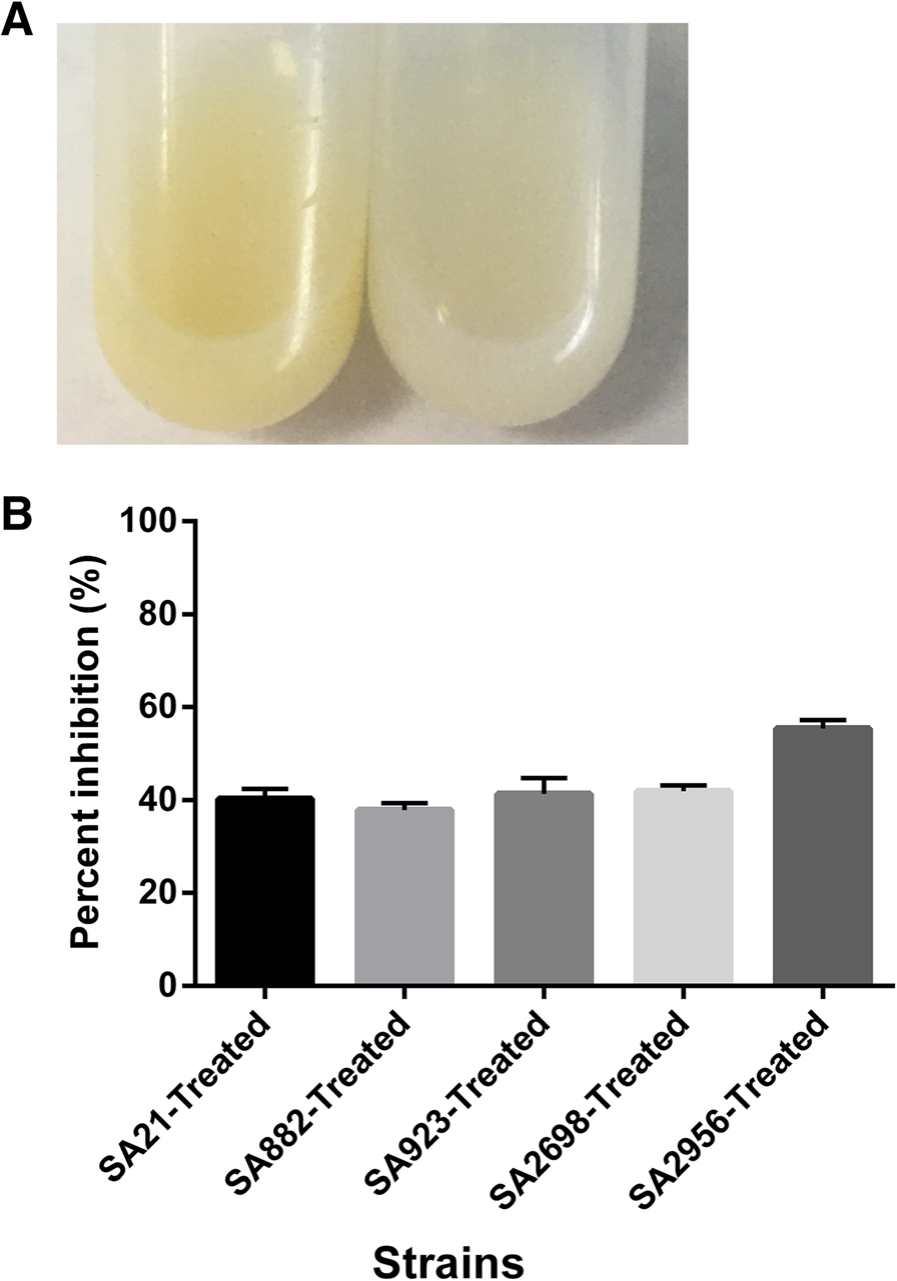
(**a**) Images showing the inhibition of pigment production in ZY-2144-treated *Staphylococcus aureus* strain SA2956. SA2956 without ZY-214-4 treatment served as control group. (**b**) The inhibitory effect of ZY-214-4 on pigment production in *S. aureus*. Images made by GraphPad Prism 6 (GraphPad Software, version 6.00, https://www.graphpad.com/)

We undertook a quantitative and qualitative assessment of pigment synthesis in ZY-214-4-treated and untreated cells. ZY-214-4 treatment markedly inhibited golden pigment production. Compared with the golden pigmentation of untreated *S. aureus*, that of *S. aureus* treated with ZY-214-4 was white or light yellow **(**Fig. 2a**)**. Quantitative analysis showed that pigment production was decreased by 38.7–41.8%, 36.8–38.9%, 39.0–43.8%, 41.1–42.8%, 54.1–56.7% in five ZY-214-4-treated clinical *S. aureus* isolates when compared with their respective untreated counterparts **(**Fig. 2b**)**.

### The effect of ZY-214-4 on the susceptibly of *S. aureus* to human blood and H_2_O_2_

**Fig. 3:**
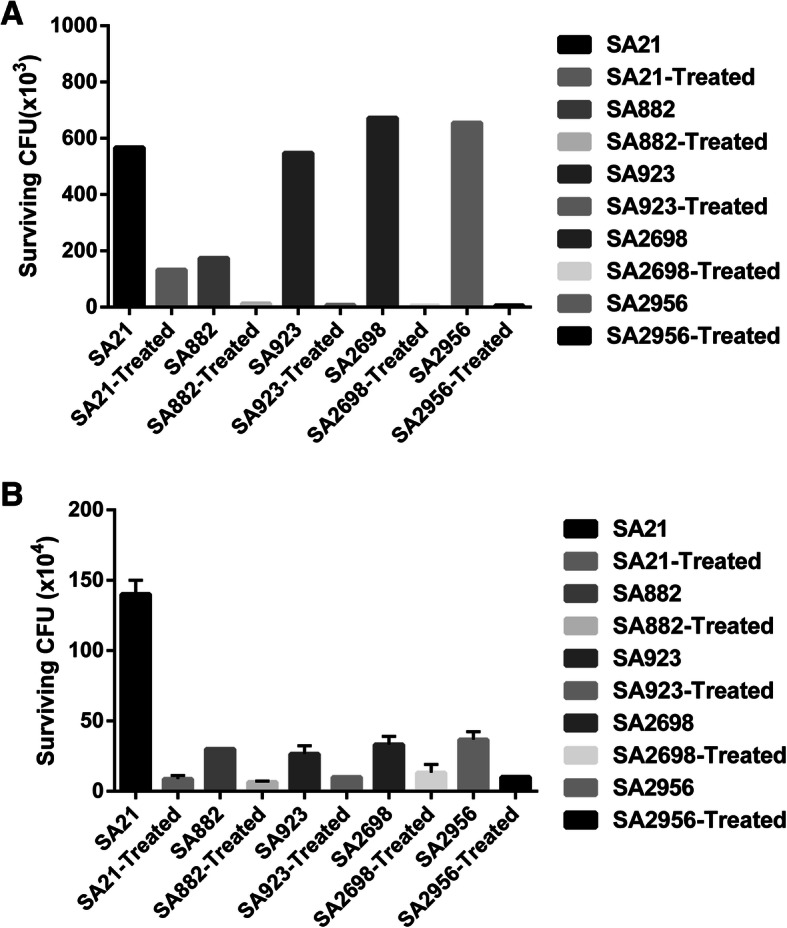
The effect of ZY-214-4 (4 μg/mL) treatment on the survival of *Staphylococcus aureus* in (**a**) H_2_O_2_ and (**b**) healthy human blood. Error bars indicate the SD and asterisks indicate statistical significance (*p* < 0.05). Images made by GraphPad Prism 6 (GraphPad Software, version 6.00, https://www.graphpad.com/)

As ZY-214-4 could inhibit pigment production in *S. aureus*, and because the pigment can shield *S. aureus* cells from host oxidants, we next compared the sensitivity of ZY-214-4-treated (4 μg/mL) and untreated *S. aureus* to H_2_O_2_ and healthy human blood. The results of an H_2_O_2_ sensitivity assay showed that ZY-214-4-treated cells were substantially more sensitive to H_2_O_2_ than untreated control cells **(**Fig. 3a**)**. Moreover, compared with untreated controls, both the number of colonies and the survival rate of clinical *S. aureus* strains were greatly decreased in the whole blood of healthy volunteers following ZY-214-4 treatment **(**Fig. 3b**)**. Together, these results indicated that ZY-214-4 treatment reduced the resistance of *S. aureus* to human blood and H_2_O_2_.

### Treatment with subinhibitory concentrations of ZY-214-4 downregulated the expression of the *sod* and *crtM* genes of *S. aureus*

**Fig. 4:**
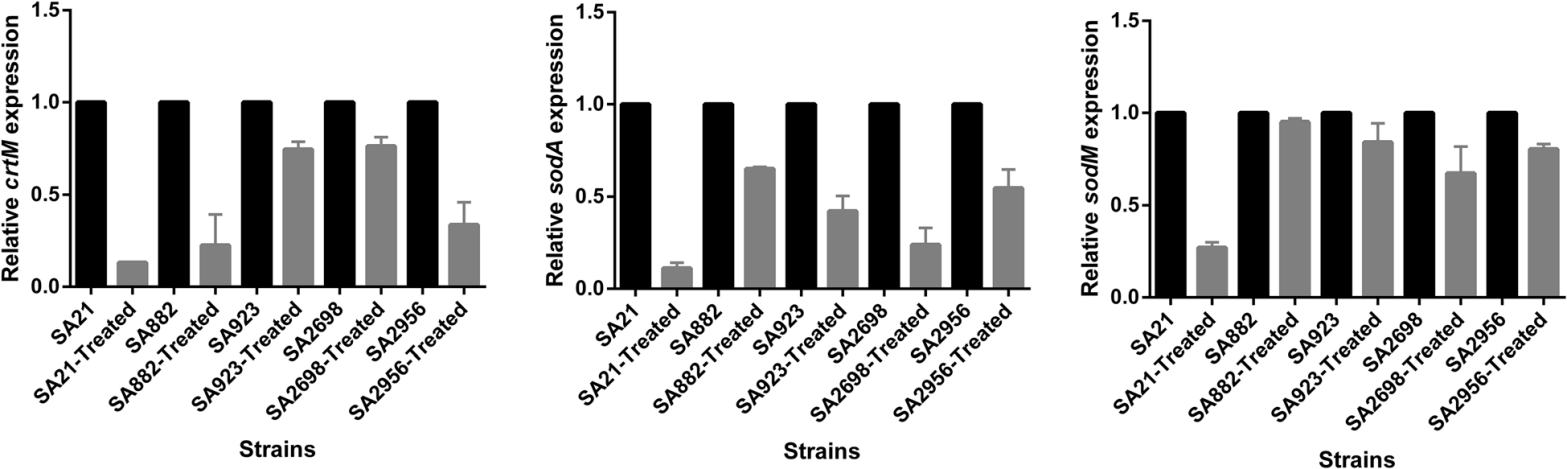
The relative expression levels of genes associated with the virulence of *Staphylococcus aureus* cultured in ZY-214-4 (4 μg/mL). Values represent means ± SD of three repeated assays. For each strain, there were significant differences when compared with the control groups (grown without ZY-214-4) (*p* < 0.05). Images made by GraphPad Prism 6 (GraphPad Software, version 6.00,https://www.graphpad.com/)

We observed that pigment synthesis was reduced in *S. aureus* and that the bacterium was more sensitive to H_2_O_2_ and healthy blood following ZY-214-4 treatment. To further explore the mechanism underlying these effects of ZY-214-4 on *S. aureus*, we used RT-qPCR to measure the expression levels of *crtM*, which is involved in antioxidant pigment synthesis, and that of *sodA* and *sodM*, which are coding for superoxide dismutase, the enzymes that scavenge oxygen free radicals and play a key role in the evasion of host defenses. We found that the expression of *crtM and sodA* were down-regulated in ZY-214-4-treated *S. aureus* cells when compared with that in controls, and 3 out 5 strains were significant for reduction in expression of *sodM*. **(**Fig. 4**)**.

### Analysis of the cytotoxicity of ZY-214-4

To evaluate the cytotoxicity of ZY-214-4, we injected silkworms with different concentrations of ZY-214-4 (2–8 μg/mL) and evaluated the effects after 24 h. No deaths were observed in either the treatment or corresponding concentration of DMSO control group (Data not shown).

### ZY-214-4 reduced the virulence of *S. aureus* in infected silkworms

**Fig. 5:**
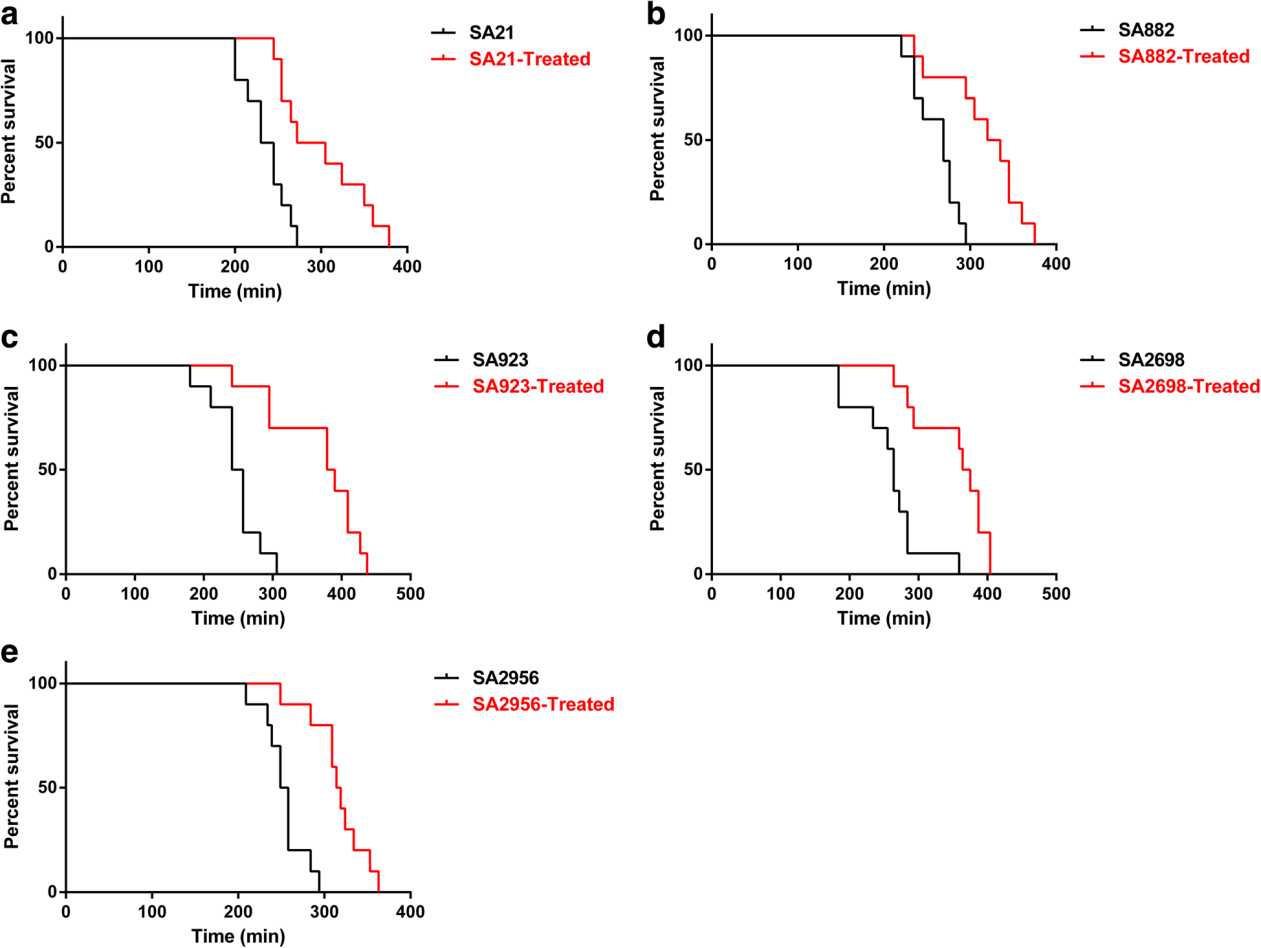
The survival of silkworm larvae inoculated with untreated or ZY-214-4-treated (4 μg/mL) *Staphylococcus aureus. p*-values < 0.05 were considered significant. (**a**) *n* = 10 per group, *p* = 0.0014. (**b**) *n* = 10 per group, *p* = 0.0010. (**c**) *n* = 10 per group, *p* = 0.0002. (**d**) *n* = 10 per group, *p* = 0.0004. (**e**) *n* = 10 per group, *p* = 0.0001. *p* < 0.05, were significantly different from that of the untreated groups. Images made by GraphPad Prism 6 (GraphPad Software, version 6.00, https://www.graphpad.com/)

We found that, in vivo, the virulence of *S. aureus* was significantly lower with ZY-214-4 treatment (4 μg/mL) than without. As shown in Fig. 5, following *S. aureus* infection, mortality occurred later in ZY-214-4-treated silkworm larvae than in untreated animals. After 5 h, the mortality rate of untreated silkworm larvae was 100% for those infected with the *S. aureus* SA21 strain, 100% for those infected with the SA882 strain, 90% for those infected with the SA923 strain, 90% for those infected with the SA2698 strain, 100% for those infected with the SA2956 strain. The respective values for ZY-214-4-treated silkworm larvae were 50, 20, 10, 30, and 30%. These results indicated that ZY-214-4 treatment can delay death in *S. aureus*-infected insects.

## Discussion

Multidrug-resistant strains of *S. aureus* are a leading cause of skin and soft tissue infection [28, 29]. The ability of *S. aureus* to survive under diverse environmental pressures is an important determinant of its pathogenicity [21, 30], highlighting the need for the development of alternative treatments. Many studies have shown that the *S. aureus* pigment is a key factor in its virulence [18, 31, 32]. The biosynthetic pathway of pigment is disrupted in a “deleted” *crtM* of *S. aureus*, resulting in the absence of pigmentation and enhanced susceptibility to killing by ROS [18]. One study reported that, in a mouse subcutaneous abscess model, *S. aureus* mutants with impaired carotenoid biosynthesis were more easily killed by oxidants and neutrophils and exhibited lower pathogenicity when compared with their wild-type counterparts [18].

In this study, we synthesized a new small-molecule compound—ZY-214-4—and selected five clinical *S. aureus* strains isolated from different sites of infection to investigate the effect of subinhibitory concentrations of ZY-214-4 on the virulence of this bacterium. Because we found that high concentrations of ZY-214-4 could inhibit *S. aureus* growth **(Additional** Figure 1**)**, we selected a subinhibitory concentration (4 μg/mL) that did not affect the growth of the bacterium, thus excluding the possibility that any reduction in virulence could result from a reduction in the number of bacteria.

The pigment of *S. aureus* has been reported to be an important virulence factor [33]. The pigment has antioxidant properties, and its many double bonds can react with ROS produced by neutrophils and macrophages, thereby protecting *S. aureus* against oxidative stress [34]. The first key step in pigment biosynthesis is catalyzed by dehydrosqualene synthase (also known as diphosphonene synthase or CrtM) [35]. Many related studies have found that there is a positive correlation between pigment production and *crtM* expression [35, 36]. Here, we found that pigment production and *crtM* gene expression were significantly downregulated in *S. aureus* under the effect of ZY-214-4. We speculate that ZY-214-4 exerts its inhibitory effect on pigment production by reducing the expression of *crtM*.

To deal with ROS, bacteria have evolved complex oxidative stress response mechanisms [37]. Notably, *S. aureus* has developed several means of escaping the immune systems of its hosts [38, 39], including phagocyte-mediated oxidative killing [40, 41]. This resistance is mediated by SOD production [42–44]. The absence of *sodA* can reduce *S. aureus* virulence in a model of abscess or retroorbital infection [45, 46]. SodM is as important as SodA [16]. SOD is a representative antioxidant enzyme that can eliminate ROS produced under oxidative stress. SOD may also help bacterial pathogens survive against oxidative outbreaks produced by inflammatory cells [47]. As *sodA* genes was downregulated in this study, the expression of *sodM* in more than half of *S. aureus* was also significantly down-regulated. We suggest that sub-bacteriostatic concentrations of ZY-214-4 can weaken the antioxidant defense of *S. aureus* by inhibiting *sod* expression. Insects possess both cellular and humoral immune response pathways, and the related literature reported that the virulence of the strain was weakened by drug action [48, 49]. In our study, we found that ZY-214-4 could reduce the virulence of *S. aureus* in the silkworm. Under the same conditions, the survival time of treated animals was significantly different from that of untreated controls.

The use of mammals for drug development is expensive and ethically problematic [50]. The mechanisms involved in the absorption, distribution, metabolism, and excretion of chemicals are similar in silkworm larvae and mammals [51, 52]. In this study, we found that ZY-214-4 was not cytotoxic within the concentration range tested, and may be beneficial for the treatment of *S. aureus* infection.

## Conclusions

In summary, we found that treatment with a subinhibitory concentration of a new small molecule, ZY-214-4, can reduce the virulence of *S. aureus* by inhibiting pigment production. This study provides a basis for exploring potential drug targets and developing new drugs for the treatment of *S. aureus* infection. However, this study also had some limitations. For example, the level of protection that ZY-214-4 provides against mortality of silkworms is not impressive. Further investigations are needed to clarify the mechanisms underlying how ZY-214-4 regulates the expression of *crtM* and *sod*.

## Methods

### Bacterial strains

The strains used in this study are listed in Table 1. The five *S. aureus* strains—SA21, SA882, SA923, SA2698, and SA2956—were isolated from patients at the First Affiliated Hospital of Wenzhou Medical University. The *S. aureus* isolates and the medical records of the patients were obtained for research purposes with the approval of the Ethics Committee of The First Affiliated Hospital of Wenzhou Medical University. Written informed consent was obtained from all the patients.

**Table 1:** The minimum inhibitory concentrations (MIC) of ZY-214-4 against five *Staphylococcus aureus* strains

Strain	MIC (μg/mL)	Ward	Year	Source	Antimicrobial Agents
SA21	64	Digital subtraction angiography (DSA)	2012	Tissue	PG(R);OX(R);EM(R);CC(R);LVX(R); MXF(R);GM(R);RIF(R)
SA882	64	Digestive ward	2014	Wound exudate	PG(R);OX(S);EM(S);CC(S);LVX(S); MXF(S);GM(S);RIF(S)
SA923	64	Neurology ward	2014	Sputum	PG(R);OX(R);EM(R);CC(R);LVX(R); MXF(R);GM(S);RIF(S)
SA2698	256	Emergency rescue	2017	Blood	PG(R);OX(S);EM(S);CC(S);LVX(S); MXF(S);GM(S);RIF(S)
SA2956	256	Hemodialysis	2017	Blood	PG(R); OX(S);EM(R);CC(R); LVX(R); MXF(R);GM(R);RIF(R)

*PG* Penicillin G; *OX* Oxacillin; *EM* Erythromycin; *CC* Clindamycin; *LVX* Levofloxacin; *MXF* Moxifloxacin; *GM* Gentamicin; *RIF* Rifampicin. R and S denotes drug resistance and drug sensitivity, respectively

### Procedure for the synthesis of C_19_H_11_BrNO_4_

**Fig. 6:**
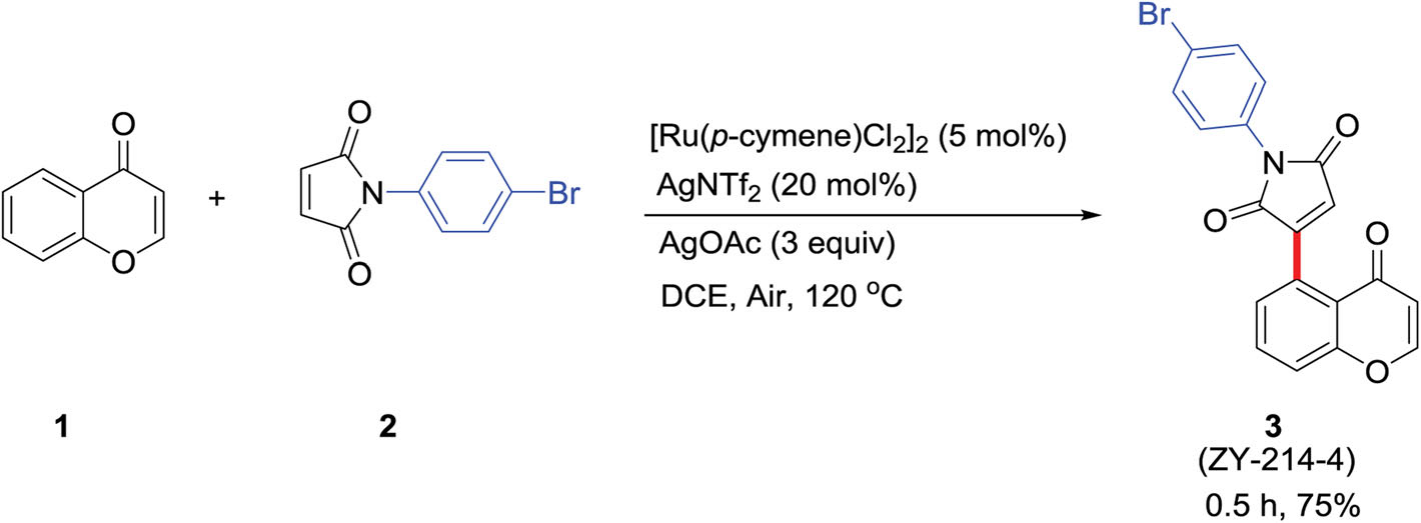
Procedure for ZY-214-4 synthesis. 1: Chromones; 2: Maleimides; 3: ZY-214-4

ZY-214-4 **(**Fig. 6**)** was synthesized by the School of Pharmacy, Wenzhou Medical University [53]. In step 1, chromone 1 (0.2 mmol, 1 equivalent) and maleimide 2 (0.5 mmol, 2.5 equivalent) were completely dissolved in 2 mL of 1,2-Dichloroethane (0.1 M DCE) in a 12-mL screw-cap tube. In step 2, [Ru(p-methylbenzyl)Cl_2_]_2_ (0.01 mmol, 0.05 equivalent), AgNTf_2_ (0.04 mmol, 0.2 equivalent), and AgOAc (0.6 mmol, 3 equivalent) were added to the reaction mixture at room temperature. For step 3, the mixture was placed on a heating mantle and the temperature was raised to 120 °C for 0.5 h, with stirring. In step 4, when the reaction was completed, the entire reaction mixture was directly loaded into a silica gel column, followed by purification with petroleum ether/EtOAc (step 5), yielding the desired product (product 3) with a yield of 75%. All the reagents used were of analytical grade **(Additional** Figure 2**)**.

### MIC determination

ZY-214-4 was dissolved in dimethyl sulfoxide (DMSO, BOYUN, SH, China) at a concentration of 20 mg/mL. The broth microdilution method based on CLSI guidelines was used to determine the minimal inhibitory concentration (MIC) [54]. The MIC was defined as the lowest concentration at which no visible bacterial growth was observed. To exclude the influence of the solvent, during the determination, we simultaneously tested the same volume of solvent as a control.

### Growth assay

The *S. aureus* strains were grown in TSB (Becton, Dickinson and Company, NJ, USA) to an optical density (OD) of 0.3 at 600 nm, following which the cultures were aliquoted into five flasks. Different doses of ZY-214-4 were then added to the culture to final concentrations of 4 μg/mL,8 μg/mL and 16 μg/mL. An Erlenmeyer flask containing only TSB was used as a blank control. All the cultures were incubated at 37 °C with shaking at 220 rpm. The OD_600_ value was measured hourly for 24 h. The assay was performed in triplicate.

### Pigment extraction

To evaluate pigment production, the five *S. aureus* strains were inoculated into 10 mL of TSB with or without ZY-214-4 (4 μg/mL). After 12 h of incubation, the cultures were centrifuged at 10,000 rpm (enppendorf, F-34-6-38) for 10 min. The pellets were washed twice with PBS, resuspended in 2 mL of methanol, and placed in an incubator for 24 h with shaking. The samples were then centrifuged at 10,000 rpm (enppendorf, F-24-6-38) for 10 min, and the OD value was measured at 465 nm. The percent inhibition of pigment production was calculated as follows: pigment inhibition rate (%) = [(ControlOD_465_ − TreatedOD_465_)/Control OD_465_] × 100 [31, 55].

### Oxidant susceptibility assay

H_2_O_2_ sensitivity assays were performed as previously described [56]. Control and ZY-214-4-treated (4 μg/mL) *S. aureus* were pelleted by centrifugation at 8000 rpm (enppendorf, F-34-6-38) for 10 min and resuspended in PBS containing 0.25% H_2_O_2_ (The chemical reagent 30% hydrogen peroxide was diluted by aseptic PBS) at 37 °C for 1 h. The cells were then serially diluted with PBS, spread on TSB agar plates, and incubated at 37 °C for 12 h. The numbers of viable cells were counted after incubation to determine whether ZY-214-4 affected *S. aureus* susceptibility to H_2_O_2_.

### Human whole-blood killing assay

For the whole-blood killing assay, cultures of each strain treated or not with ZY-214-4 (4 μg/mL) were centrifuged and resuspended in sterile PBS to a final concentration of 1 × 10^7^ CFU/mL. Whole blood from healthy human volunteers was collected into Vacutainer PT tubes (Becton, Dickinson and Company, NJ, USA). Aliquots (600 μL) of whole blood were transferred into 1.5-mL test tubes and mixed with 200 μL of bacterial samples to a final concentration of 2.5 × 10^6^ CFU/mL as previously described [57]. The tubes were incubated at 37 °C with shaking (250 rpm) for 1 h, following which dilutions were spread on Colombian blood plates to count the numbers of colonies.

### RNA-seq and identification of differentially expressed genes

Bacteria were cultured for 12 h in TSB with or without ZY-214-4 (4 μg/mL) and then collected by centrifugation at 12,000×*g* for 1 min at 4 °C. RNA was extracted using the QIAGEN RNeasy Maxi Kit (QIAGEN, BER, Germany) following the manufacturer’s instructions. The RNA was sequenced using the Illumina HiSeq X platform with a paired-end read length of 150 bp. DEGseq software [58] was used to analyze the effect of ZY-214-4 on gene expression. Differences in gene expression were considered significant with |log2 (fold change)| > 1 and *p* < 0.005.

### Quantitative real-time RT-PCR

*S.aureus* was cultured in the medium with and without ZY-214-4 (4 μg/mL). After 12 h, RNA was extracted as described above. The primer pairs used for qPCR are listed in Table 2. Total RNA was reverse transcribed using a Takara RNA PCR Kit (Takara, Tokyo, Japan). qPCRs were performed in 20-μL reaction mixtures using Luna Universal qPCR Master Mix (New England Biolabs, MA, USA). Each test was performed independently in triplicate.

**Table 2:** Primers used for RT-qPCR

Primer name	Sequence (5′–3′)
*gyrb*-RT-F	ACATTACAGCAGCGTATTAG
*gyrb*-RT-R	CTCATAGTGATAGGAGTCTTCT
*sodA*-RT-F	GACAGACATCATAACACTTA
*sodA*-RT-R	ACTCCCAGAATAATGAATG
*sodM*-RT-F	CTGTACCTTCTACTGCAGCATTTA
*sodM*-RT-R	TTAGAACCACATTTTGACAAAGAA
*crtM*-RT-F	CATCGTATGTCTGATGTG
*crtM* -RT-R	GCTGAATTATTCGGATATTG

### Assessment of the toxicity of ZY-214-4 in the silkworm

The toxicity of ZY-214-4 against the silkworm was assessed as previously described, with slight modifications [59]. A disposable plastic syringe (Terumo, TY, Japan) was used to inject different concentrations (2–8 μg/mL) of ZY-214-4 (0.05 mL) into the body of silkworm larvae. The survival rate was measured one day after injection.

### The infection of silkworm larvae for the assessment of *S. aureus* virulence following ZY-214-4 treatment

*Staphylococcus aureus* strains were cultured on Columbia blood agar plates at 37 °C overnight. The next day, *S. aureus* was inoculated into TSB and grown to the logarithmic phase at 37 °C with shaking (220 rpm). ZY-214-4 was added to a final concentration of 4 μg/mL. A bacterial solution without ZY-214-4 was used as control. After 12 h, the bacteria were collected by centrifugation at 8000 rpm for 5 min at 4 °C, washed three times with phosphate-buffer saline (PBS), and diluted to 0.5 McFarland standard at 600 nm. The total colony units were further adjusted to obtain the required dose. For the infection of silkworm larvae, there were 10 larvae in each group, and the weight of each larva is 250 mg. Injection was performed as previously described [60] with slight modifications. In brief, a syringe was used to inject 50 μL of *S. aureus* into the last left forelimb of each larva. After the injection, the larvae were placed in an incubator at 37 °C, and larval mortality was recorded. Larvae were considered to be dead when they did not respond to touch. Silkworm larvae that were not exposed to ZY-214-4 and those injected with phosphate-buffered saline (PBS) were used as controls.

### Statistical analysis

GraphPad Prism 6 (GraphPad Software, version 6.00, https://www.graphpad.com/) was used to analyze the experimental data. A *p*-value < 0.05 was considered statistically significant. In addition to using log rank test analysis of survival rate of silkworm, all others used one-way analysis of variance.

## Supplementary Information

**Additional file 1 **Figure 1 Growth curves for *Staphylococcus aureus* strains cultured with ZY-214-4(4 μg/mL). TSB was used as a blank control. Images made by GraphPad Prism 6 (GraphPad Software, version 6.00, https://www.graphpad.com/).

**Additional file 2.** Figure 2 HPLC of ZY-214-4.

## Data Availability

The datasets generated during the current study are available from the corresponding author upon reasonable request. Most of the data is included in this published article.
