# Supplementary figures and images for: The small molecule ZY-214-4 may reduce the virulence of Staphylococcus aureus by inhibiting pigment production
Source: BMC Microbiol. 2021 Feb 27;21:67. doi: 10.1186/s12866-021-02113-5 (PMC7916275; doi:10.1186/s12866-021-02113-5)

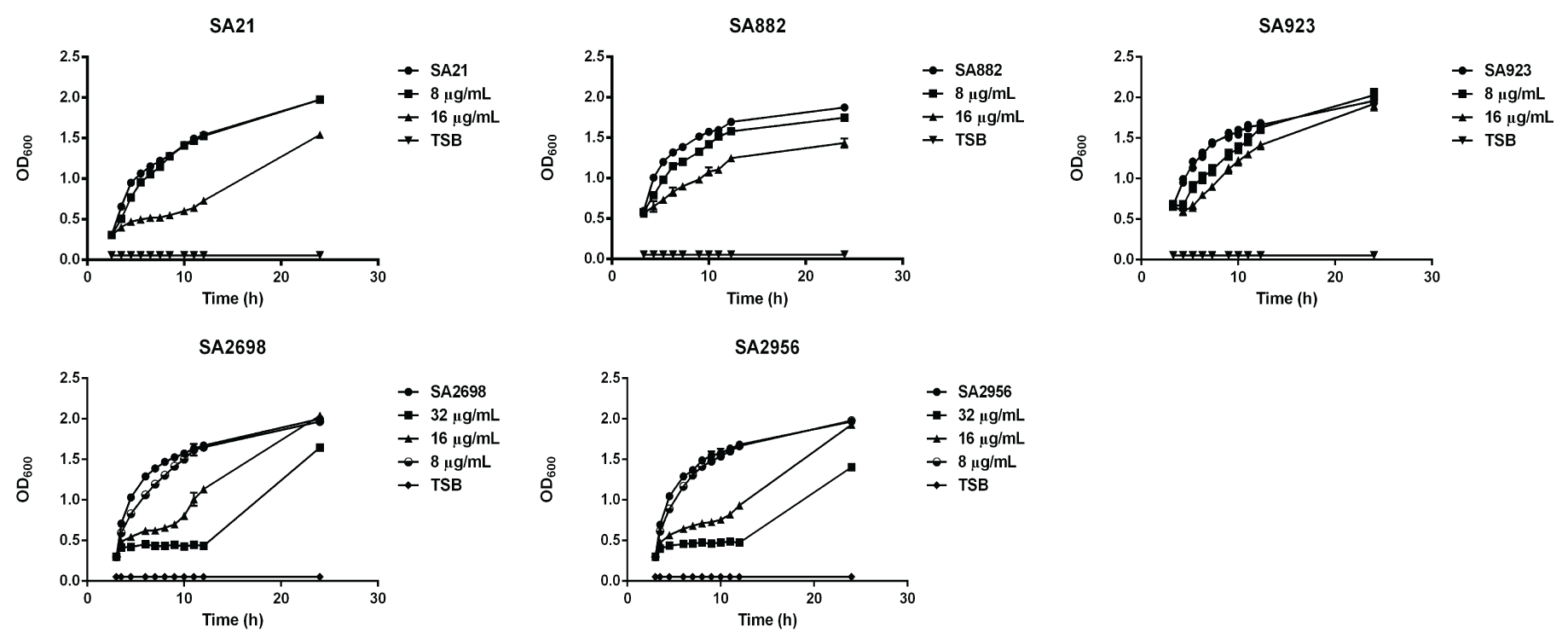

Supplement: Supplementary file 1 — Additional file 1 Figure 1 Growth curves for Staphylococcus aureus strains cultured with ZY-214-4(4 μg/mL). TSB was used as a blank control. Images made by GraphPad Prism 6 (GraphPad Software, version 6.00, https://www.graphpad.com/). [file 12866_2021_2113_MOESM1_ESM.tif]

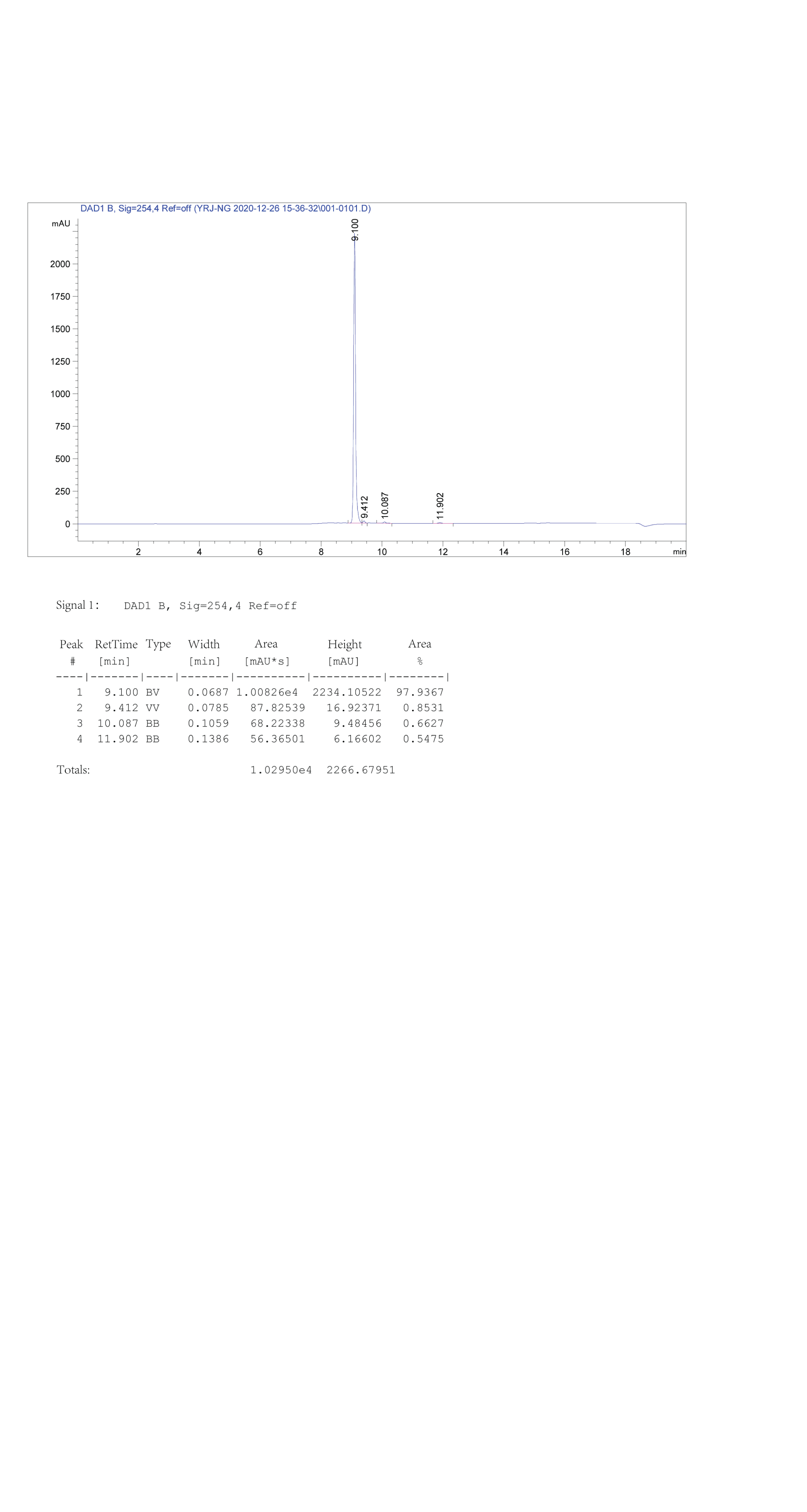

Supplement: Supplementary file 2 — Additional file 2. Figure 2 HPLC of ZY-214-4. [file 12866_2021_2113_MOESM2_ESM.tif]
